# Subgrading of G2 Pancreatic Neuroendocrine Tumors as 2A (Ki67 3% to < 10%) Versus 2B (10% to ≤ 20%) Identifies Behaviorally Distinct Subsets in Keeping with the Evolving Management Protocols

**DOI:** 10.1245/s10434-024-15632-y

**Published:** 2024-07-02

**Authors:** Ozgur Can Eren, Pelin Bagci, Serdar Balci, Nobuyuki Ohike, Burcu Saka, Cenk Sokmensuer, Can Berk Leblebici, Yue Xue, Michelle D. Reid, Alyssa M. Krasinskas, David Kooby, Shishir K. Maithel, Juan Sarmiento, Jeanette D. Cheng, Orhun Cig Taskin, Yersu Kapran, Zeynep Cagla Tarcan, Claudio Luchini, Aldo Scarpa, Olca Basturk, N. Volkan Adsay

**Affiliations:** 1 Department of Pathology, Koç University, Koç University Hospital, Istanbul, Türkiye; 2 Department of Pathology, Marmara University, Istanbul, Türkiye; 3 Department of Pathology, Memorial Sisli Hospital, Istanbul, Türkiye; 4 Department of Pathology, St. Marianna University, Kawasaki, Kanagawa Japan; 5 Department of Pathology, Hacettepe University, Ankara, Türkiye; 6 Department of Pathology, Northwestern University, Evanston, IL USA; 7 Department of Pathology, Emory University, Atlanta, GA USA; 8 Department of Surgery, Emory University, Atlanta, GA USA; 9 Department of Pathology, Piedmont Hospital, Atlanta, GA USA; 10 Department of Pathology, Memorial Sloan Kettering Cancer Center, New York, NY USA; 11 Department of Diagnostics and Public Health, Section of Pathology and ARC-Net Research Centre, University of Verona, Verona, Italy

**Keywords:** Pancreatic neuroendocrine tumor, Ki-67 proliferative index, Grade, Metastasis

## Abstract

**Background:**

Grade 1/2 PanNETs are mostly managed similarly, typically without any adjunct treatment with the belief that their overall metastasis rate is low. In oncology literature, Ki67-index of 10% is increasingly being used as the cutoff in stratifying patients to different protocols, although there are no systematic pathology-based studies supporting this approach.

**Methods:**

Ki67-index was correlated with clinicopathologic parameters in 190 resected PanNETs. A validation cohort (*n* = 145) was separately analyzed.

**Results:**

In initial cohort, maximally selected rank statistics method revealed 12% to be the discriminatory cutoff (close to 10% rule of thumb). G2b cases had liver/distant metastasis rate of almost threefold higher than that of G2a and showed significantly higher frequency of all histopathologic signs of aggressiveness (tumor size, perineural/vascular invasion, infiltrative growth pattern, lymph node metastasis). In validation cohort, these figures were as striking. When all cases were analyzed together, compared with G1, the G2b category had nine times higher liver/distant metastasis rate (6.1 vs. 58.5%; *p* < 0.001) and three times higher lymph node metastasis rate (20.5 vs. 65.1%; *p* < 0.001).

**Conclusions:**

G2b PanNETs act very similar to G3, supporting management protocols that regard them as potential therapy candidates. Concerning local management, metastatic behavior in G2b cases indicate they may not be as amenable for conservative approaches, such as watchful waiting or enucleation. This substaging should be considered into diagnostic guidelines, and clinical trials need to be devised to determine the more appropriate management protocols for G2b (10% to ≤ 20%) group, which shows liver/distant metastasis in more than half of the cases, which at minimum warrants closer follow-up.

Neuroendocrine neoplasms in the pancreas occur in two entirely distinct groups recognized based on the morphologic grounds, as poorly differentiated carcinomas (PDNECs, which are essentially pancreatic counterparts of high-grade small-cell and large-cell neuroendocrine carcinomas in the lung) and the well-differentiated pancreatic neuroendocrine tumors (PanNETs; i.e., what used to be called islet cell tumors/carcinomas, or pancreatic counterparts of what used to be called “carcinoid”).^1,2^ While PDNECs are rapidly fatal malignancies, PanNETs are widely regarded as low-grade neoplasms with relatively limited metastatic potential to an extent that watchful waiting is considered for smaller examples.^3,4^ However, it is being recognized increasingly that a subset of PanNETs develop metastases and behave aggressively, clearly not as dismal as PDNECs but nevertheless often with dissemination and fatality.^4^ Some of these patients present to oncologists and skip surgeons’ attention and thus create some disparate views on the nature of PanNETs in respective literature. One parameter that could potentially help to identify this aggressive subset is the grade based on Ki67 proliferation index. It is now fairly well appreciated that those rare grade 3 PanNETs, defined as Ki67 >20% (which had been previously classified together with PDNECs until WHO-2017 when they were moved to PanNET category) have substantial potential for aggressive behavior (albeit nowhere near as bad as PDNECs^4–6^), and adjuvant therapy is typically considered in their management.^7–9^ In contrast, however, currently, G1/G2 (G1 Ki67 < 3%; and G2 Ki67 3–20%) PanNETs are widely viewed as benign-behaving tumors, and in fact those smaller than 2 cm are typically managed by watchful waiting without resection, regardless of the grade.^10,11^ Along these lines, for resected G1/G2 PanNETs, current management guidelines do not have any specific recommendation other than follow-up. However, as mentioned previously, some of these ultimately develop metastases and exhibit aggressive behavior and which cases are prone to this is currently not clear.^3,4^

In order to detect the G1/G2 PanNETs that are more likely to exhibit aggressive behavior, there have been efforts to identify histopathologic parameters to help guide the management. Recently, some morphologic variants, such as those with more abundant cytoplasm, single, prominent nucleoli (oncocytic and hepatoid; “metabolic cell phenotype”), and diffuse growth were found to be significantly more aggressive than the more organoid (ductulo-insular and paraganglioma-like) or degenerative (symplastic/pleomorphic) examples.^12–14^ Another parameter associated with more aggressive behavior was the more scirrhous growth pattern,^15^ infiltration pattern at the edges for which a classification system with strong prognostic correlation was proposed and proved to be even more valuable than grade, which is supported by other studies.^16,17^ However, as these recently proposed classification schemes are being verified and fine-tuned for incorporation into guidelines, the grade and stage remain as almost the sole tool in resected PanNETs to help guide the subsequent management.

It is under question whether Ki67 proliferation index is being utilized as effectively in the prognostication of G1/2 PanNETs. For example, although recent studies emphasized the usage of more reliable counting methodologies and although “eye-balling” approach is discouraged per consensus manuscripts, it is still being used widely in daily practice.^18–23^ Moreover, the current cutoffs, which were initially determined based on limited number of cases, and mostly extrapolated from the literature on gastrointestinal NETs (previously called “carcinoids”) are now widely acknowledged to have limitations in clinical application for PanNETs.

Recently, mostly in the oncology literature, patients with Ki67 ≥ 10% are increasingly being viewed as the group that warrant further attention, close follow-up, and consideration for somatostatin analog (SSA)/peptide receptor radionuclide therapy (PRRT) and/or even conventional chemotherapy.^24–35^ Although this management approach is mostly based on the Ki67 index on metastatic tumors, it also is being considered for resected PanNETs, yet the literature supporting this approach from pathology perspective is limited.^36^

In this study, we investigated the relevance and clinicopathologic associations of cutoff of 10% in a cohort of 190 PanNETs and subsequently tested its significance in a separate cohort of 145 cases. We found, in all these cohorts, that indeed the subset of G2 PanNETs that have Ki67 10% to ≤ 20%, which we propose to be documented as G2b, has significantly more aggressive characteristics, including incomparably higher rate of liver metastasis, and as such deserve to be notated separately in alliance with the developing management algorithms that are already placed in practice, although not yet in guidelines.

## Materials and Methods

The study construct was reviewed and approved by the institutional review boards of the participating institutions.

### Case Selection

For the initial cohort of the study, pathology archives were searched, and the resected PanNET cases encountered in Marmara University, Hacettepe University and Koç University, Türkiye, and Wayne State University and Emory University, USA, were retrieved. Based on the results of the initial analysis, a separate cohort from Verona University, Italy, and Memorial Sloan Kettering Cancer Center, NY, also were retrieved and analyzed as the validation cohort.

Demographic information was obtained from original pathology reports and verified from the databases of the institutes when available. Survival data were collected from hospital records and national health records database.

Poorly differentiated neuroendocrine carcinomas and ambiguous cases in which a clear NET/NEC distinction could not be made were not included.^4,6,37–39^ Also, carefully excluded were cases with mixed acinar-neuroendocrine neoplasm and cases of other nonneoplastic and neoplastic (acinar carcinomas, pancreatoblastomas, solid-pseudopapillary tumors) mimickers of PanNETs.^12,40,41^

### Ki67 Subgrouping

In the primary cohort, for each case, all slides were reexamined by the authors. Ki67 analysis was performed as described in detail previously.^18^ Briefly, more cellular and atypical foci with mitotic activity were selected for Ki67. Ki67 stained slides were scanned at 10x objective, and the hot spot areas were identified. Camera captured images of the hotspot field were printed and cells were counted manually: tumor cells with nuclear Ki67 staining were circled, while unstained tumor cells were crossed off. Nuclear labeling in the nontarget cells (endothelial cells, lymphocytes, neutrophils, and macrophages) were carefully excluded.

For the validation cohort, Ki67 index was extracted from the surgical pathology reports; a separate count was not performed by the authors in this cohort.

Cases with Ki67 < 3% (including cases with 2.99 but not 3) were classified as G1. The WHO G2 group (Ki67 3–20%) also was evaluated in two subgroups as G2a (Ki67 3% to <10%) and G2b (Ki67 10% to ≤ 20%). Cases with Ki67 > 20% were regarded as G3. As mentioned previously, poorly differentiated neuroendocrine carcinomas were excluded.

### Clinicopathologic Parameters

Age at diagnosis and sex were obtained for each case. Tumor size, vascular invasion, and perineural invasion were reevaluated by the authors along with the lymph node metastasis status in the resection specimens. Distant metastasis status was retrieved from the patient medical records. Tumor staging was performed according to the AJCC.^42^

In addition, in the primary cohort, for each PanNET, the invasiveness pattern at the periphery was evaluated as “non/minimally infiltrative” (NI), “moderately infiltrative” (MI), and “highly infiltrative” (HI) groups, as previously described^16^ to assess the association of the proposed grading system with this parameter.

Follow-up data concerning clinical, radiological, or histopathologic evidence of tumor recurrence and survival data were extracted from patient records of the institutions and the national electronic databases.

### Correlative Analysis

Correlations between Ki67 index grade groups and various conventional established clinicopathologic parameters of aggressiveness (tumor size, vascular invasion (VI), perineural invasion (PNI), lymph node metastasis, liver/distant metastasis) were analyzed in all cohorts. In the primary cohort, the findings were also correlated with the infiltration-based prognostic categories, a histopathologic classification scheme that recently has been to correlate with aggressive behavior. Tumors in the group < 2 cm in size were investigated separately for clinical purposes because this subset of PanNETs are now commonly placed in watchful waiting category.^10,11^

### Statistical Analysis

Descriptive statistics were presented to define continuous variables. The normality of continuous variables was investigated by Shapiro-Wilk’s test. The χ^2^ test was used for categorical variables along with Fisher exact test when applicable. Logistic regression was used to evaluate the effect of independent variables (which are found statistically significant at univariate analysis) on a dependent variable. Statistical significance was accepted when *p* < 0.05. Separately, to determine the true numerical cutoff that identifies the more aggressive group, the “maximally selected rank statistics” test was performed. All analysis was done with *Jamovi*^43^ and *R.*^44^

## Results

### General Characteristics

1. Initial (primary) cohort (*n* = 190)

Among 190 PanNET cases included in the primary cohort, the mean age at initial resection was 54.5 years (range 17–84), with a female:male ratio of 1.08. Mean tumor size was 3.5 (range 0.5–12.0, median 3.0) cm. With reference to AJCC 8th edition (2017), the percentages of pT1, pT2, pT3, and pT4 cases were 33%, 35%, 31%, and 1% respectively.

**Fig. 1:**
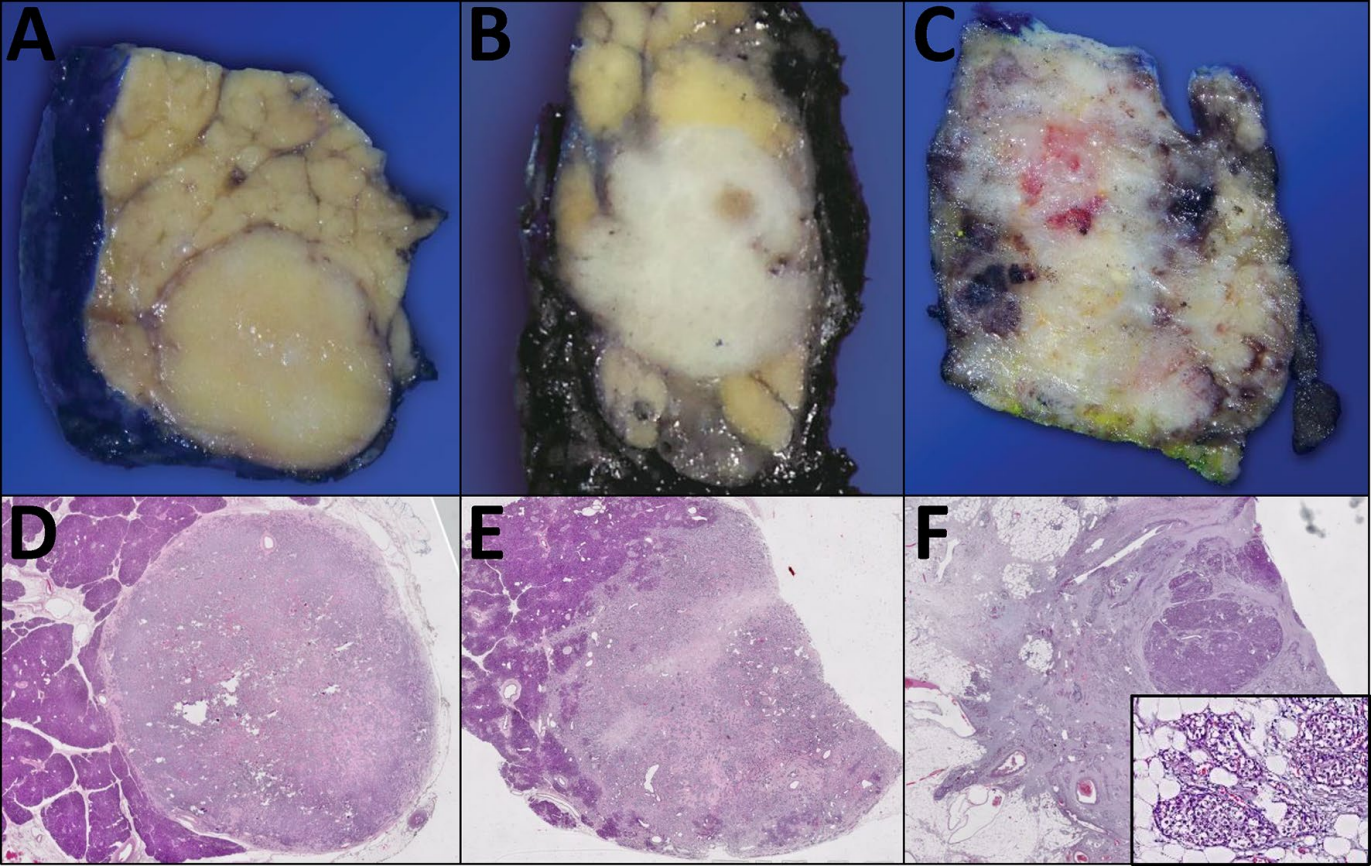
Macroscopic and microscopic correlations of categories non/minimal infiltrative (**A, D**); moderately infiltrative (**B, E**), and highly infiltrative (**C, F**)

Regarding the infiltration pattern scoring at the advancing edge (assessed in the primary cohort only), 42% of PanNETs were classified as non/minimally infiltrative (NI), 37% as moderately infiltrative (MI), and 21% as highly infiltrative (HI) (Fig. 1).

As the significant proportion of the cases were surgical referrals and case identification was conducted mostly through the pathology databases, the functionality status of the cases may not have been reliably documented in each case. In the primary cohort, 13 cases were recorded to have functionality, and these were insulinomas (*n* = 7), gastrinomas (*n* = 4), glucagonoma (*n* = 1), and somatostatinoma (*n* = 1). Thus, the majority of the cases were nonfunctional per the limited pathology records and clinical records available to the authors, as is the case in most studies nowadays.

Median follow up time was 40 (range 18–95) months. Local (lymph node) and liver-distant metastasis rates at the time of resection were 34% and 22%. During follow-up, 18 patients died from disease-related causes.

2. Validation cohort (*n* = 145)

The mean age at initial resection was 54.4 (range 30–77) years, with a female:male ratio of 0.88. Mean tumor size was 3.6 (range 0.7–25, median 2.8) cm. The percentages of pT1, pT2, pT3, and pT4 cases were 25%, 36%, 36%, and 3%, respectively. Median follow up time was 72 (range 0.5–276) months. Local (lymph node) and liver/distant metastasis rates at the time of resection were 41% and 30%. During follow-up, 11 patients died from disease-related causes.

### Ki67 Grades: Frequency and Correlation with Clinicopathologic Parameters

**Fig. 2:**
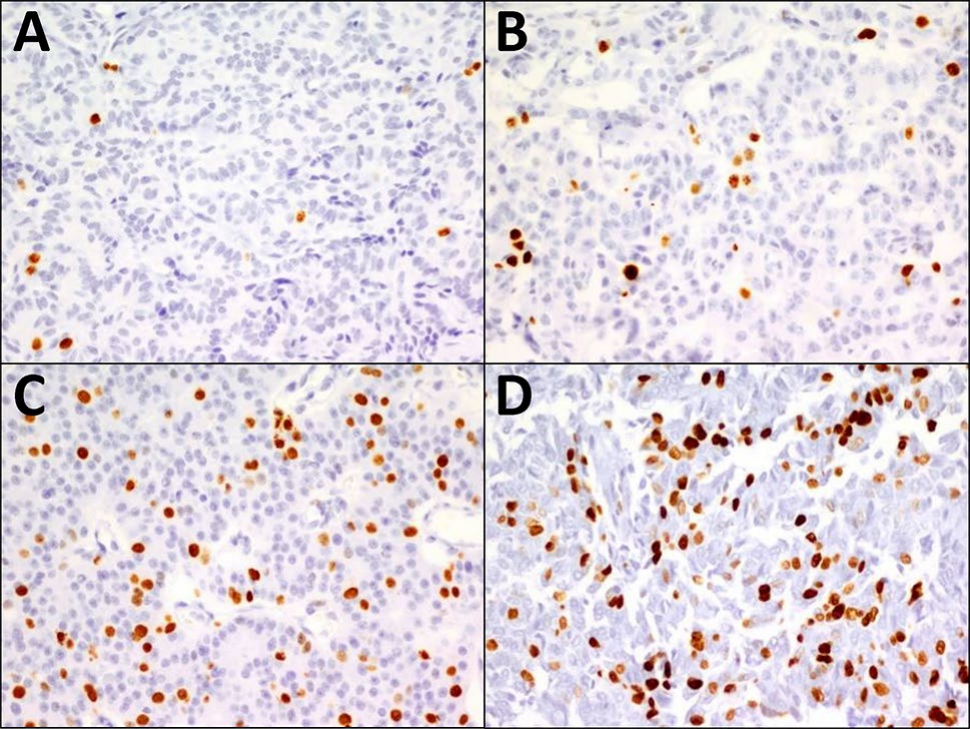
PanNETs with Ki67 index 1.2% (**A**), 5.6% (**B**), 12.4% (**C**), and 29.4% (**D**) corresponding to G1, G2a, G2b, and G3, respectively

The mean Ki67 index was 6.84% in primary, 7.45% in validation, and 7.20% in combined cohorts. The distribution of cases into grades G1, G2a, G2b, and G3 were as follows for the primary cohort: G1 48.4% (*n* = 92), G2a 36.9% (*n* = 70), G2b 7.9% (*n* = 15), and G3 6.8% (*n* = 13). For the validation cohort, these figures were G1 35.2% (*n* = 51), G2a 37.9% (*n* = 55), G2b 19.3% (*n* = 28), and G3 7.6% (*n* = 11). Please see Table 1 for comparison of cohorts, and Fig. 2 for representative Ki67 examples from each category.

**Table 1:** Comparison of primary and validation cohorts

	Primary cohort	Validation cohort	Entire cohort	Primary vs. validation cohort, *p*-values
*n*	190	145	335	
Mean age, year (SD)	54.5 (11.5)	56.4 (15)	55.3 (13.7)	0.212
F/M	1.09	0.88	1.01	0.311
T3 and T4 stage (%)	32.2	39.3	35.1	0.170
Median size, cm	3.00	2.80	3.50	0.695
LN metastasis (%)	33.9	40.7	37	0.209
Presence of PNI (%)	41.7	29.1	34.3	**0.015**
Presence of VI (%)	46.1	57.2	50.7	**0.040**
Mean Ki67	6.84	7.45	7.2	0.519
*Grade (%)*				**0.008**
G1	48.4	35.2	42.7	
G2a	36.9	37.9	37.3	
G2b	7.9	19.3	12.8	
G3	6.8	7.6	7.2	
Adverse outcome (%)	23.9	31.7	27.2	0.106

Bold values indicate statistical significance: *p* < 0.05

*LN* Lymph node, *PNI* Perineural invasion, *VI* Vascular invasion, *Adverse outcome* liver/distant metastasis + disease-related death

In 64 cases in the primary cohort, the Ki67 index rendered in the original pathology reports had not been performed by the authors (and not with the camera-captured/printed image (CCPI) method described) but rather counted by simple eye-balling by other pathologists. The comparison of the indices in the original report versus those elicited in the study in the same patients revealed the median difference to be 1.37 and mean 2.83. In 18 cases (28%), the final grade was different between the original report and the count performed for the study. However, none of the cases jumped by two categories in the subgraded approach proposed (i.e., none of the cases changed from G1 vs. G2b or G2a to G3). In 46 cases, grade did not differ between the original report and CCPI counting.

For conventional findings of aggressiveness, including PNI, VI, and infiltration score, the G2b category showed significantly higher rates than the G2a (Table 2). More importantly, for lymph node metastasis, the rate in G2b as opposed to G2a, this figure was double in the primary cohort (73.3 vs. 35.5%, *p* = 0.003), and significantly higher also in the validation (60.7 vs. 47.3%, *p* < 0.001), and combined (65.1 vs. 41.7%, *p* < 0.001) cohorts. For liver/distant metastasis, it was almost triple in the primary cohort (61.5 vs. 22.6, *p* = 0.001), almost double, in the validation (57.1 vs. 29.1, *p* < 0.001), and combined (58.5 vs. 26.1, *p* < 0.001) cohorts (Table 2).

**Table 2:** Correlation of Ki67 grades with clinicopathologic features of aggressiveness

Primary cohort (*n* = 190)					
	G1 (Ki67 < 3%)	G2a (Ki67 3 to < 10%)	G2b (Ki67 10 to ≤ 20%)	G3 (Ki67 > 20%)	*p*
*n* (%)	92 (48.4%)	70 (36.9%)	15 (7.9%)	13 (6.8%)	
Mean age, year (SD)	56.4 (14.0)	52.5 (16.0)	51.6 (14.6)	52.4 (19.1)	0.331
F/M	1.2	1.1	2	0.4	0.262
T3 and T4 stage (%)	18.9%	41.8%	46.7%	61.5%	**0.001**
Median size, cm	2.6	4.0	4.3	4.9	**0.014**
Lymph node metastasis (%)	23.7%	35.5%	73.3%	36.4%	**0.003**
Liver/distant metastasis (%)	7.5%	22.6%	61.5%	45.5%	**< 0.001**
Presence of PNI (%)	16.5%	35.7%	53.5%	46.2%	**0.002**
Presence of VI (%)	23.2%	60.0%	80.0%	92.3%	**< 0.001**
Moderate/high IPS Score (%)	46.2%	67.3%	84.6%	77.7%	**< 0.001**

Validation cohort (*n* = 145)					
	G1 (Ki67 < 3%)	G2a (Ki67 3 to < 10%)	G2b (Ki67 10 to ≤ 20%)	G3 (Ki67 > 20%)	*p*
*n* (%)	51 (35%)	55 (38%)	28 (19%)	11 (8%)	
Mean age, year (SD)	58.6 (9.59)	55.5 (13.4)	56.6 (8.91)	49.8 (13.7)	0.192
F/M	1.3	0.7	0.6	1.2	0.234
T3 and T4 stage (%)	11.8%	45.5%	64.3%	72.7%	**< 0.001**
Median size, cm	2.25	2.95	4.10	4	**0.004**
Lymph node metastasis (%)	15.7%	47.3%	60.7%	72.7%	**< 0.001**
Liver/distant metastasis (%)	3.9%	29.1%	57.1%	90.9%	**< 0.001**
Presence of PNI (%)	11.8%	49.1%	70.4%	72.7%	**< 0.001**
Presence of VI (%)	31.4%	58.2%	89.3%	90.9%	**< 0.001**

Entire cohort (*n* = 335)					
	G1 (Ki67 < 3%)	G2a (Ki67 3 to < 10%)	G2b (Ki67 10 to ≤ 20%)	G3 (Ki67 > 20%)	*p*
*n* (%)	143 (42.7%)	125 (37.3%)	43 (12.8%)	24 (7.2%)	
Mean age, year (SD)	57.2 (12.6)	53.8 (15)	54.9 (11.3)	51.2 (16.6)	0.093
F/M	1.3	0.9	0.9	0.7	0.352
T3 and T4 stage (%)	16.3%	44.2%	58.1%	66.7%	**< 0.001**
Median size, cm	2.15	3.50	4	4.8	**< 0.001**
Lymph node metastasis (%)	20.5%	41.7%	65.1%	54.5%	**< 0.001**
Liver/distant metastasis (%)	6.1%	26.1%	58.5%	68.2%	**< 0.001**
Presence of PNI (%)	14.8%	42.3%	64.3%	58.3%	**< 0.001**
Presence of VI (%)	26.2%	59.3%	86%	91.7%	**< 0.001**

Bold values indicate statistical significance; *p* < 0.05

*PNI* Perineural invasion, *VI* Vascular invasion, *IPS* Infiltration pattern score

Of note, in all these parameters, the G2b category was not only very different than the G2a but also was very similar to the G3 category (and in fact, in the primary cohort, showed numbers even higher than the G3 group, although this unexpected profile was not present in the validation cohort and mostly disappeared when all cases were combined).

Most importantly, compared with the G1 group, the proposed G2b category had incomparably higher rates of aggressiveness features, including the liver/distant metastasis rate (7.5% vs. 61.5% in primary, 3.9% vs. 57.1% in validation, 6.1% vs. 58.5% in combined cohorts).

### Tumors < 2 cm and tumors 2–4 cm

With primary and validation cohorts combined, the differences between G2b and G2a persisted when they were size matched as well. Of the 102 PanNETs that were < 2 cm in size, 65.7% (*n* = 67) were stratified as G1, 24.5% (*n* = 25) as G2a, 6.9% (*n* = 7) as G2b, and 2.9% (*n* = 3) as G3. This group demonstrated statistically significant correlation between higher grade and lymph node metastasis (15.5% for G1; 9.1% for G2a; and 57.1% for G2b, *p* = 0.020) and liver/distant metastasis (1.6% for G1, 8.7% for G2a, 50% (*n* = 3/6) for G2b, *p* < 0.001).

Of the 94 PanNETs that were 2 to 4 cm in size, 50% (*n* = 47) were stratified as G1, 34% (*n* = 32) as G2a, 10.6% (*n* = 10) as G2b, and 5.3% (*n* = 5) as G3. In this group, the rate of lymph node metastasis (20.9% for G1; 48.3% for G2a; and 80% for G2b, *p* = 0.003) and liver/distant metastasis (6.8% for G1; 16.7% for G2a; 70.0% for G2b) increased progressively.

### Maximally Selected Rank Statistics and Odds Ratios

The “maximally selected rank statistics” analysis performed to determine the main cutoff to predict the adverse outcome in the primary cohort, where Ki67 count was performed with the CCPI method, identified this number as Ki67 index of 12%.

To establish the correlation of conventional risk factors with adverse events (liver/distant metastasis or disease related death), multivariate analysis was performed. In this analysis, the current grading system of G1-G3 was not found to be significant, whereas the proposed grading was found to be independent along with tumor stage and lymph node metastasis. PNI and VI did not reach statistical significance (Table 3).

**Table 3:** Odds ratios of adverse outcomes (liver/distant metastasis + disease related death) per clinicopathological features for primary, validation, and entire cohorts

		Primary cohort (*n* = 190)			Validation cohort (*n* = 145)			Entire cohort (*n* = 335)		
		*p*	OR	95% CI	*p*	OR	95% CI	*p*	OR	95% CI
Age		0.752	1.00	0.97–1.03	0.072	0.96	0.92–1.00	0.190	0.98	0.96–1.01
Sex	Female									
	Male	0.745	0.86	0.34–2.13	0.750	1.19	0.41–3.40	0.973	0.99	0.52–1.88
Tumor stage	T1–T2									
	T3–T4	**0.009**	3.48	1.37–9.07	**0.031**	3.29	1.13–10.13	**< 0.001**	3.53	1.80–7.04
PNI	Absent									
	Present	0.373	1.58	0.57–4.34	0.122	0.38	0.10–1.23	0.877	0.94	0.45–1.93
VI	Absent									
	Present	0.705	0.79	0.23–2.58	**0.013**	5.88	1.56–26.97	0.261	1.59	0.71–3.57
Lymph node metastasis	Absent									
	Present	**0.011**	3.68	1.37–10.32	0.058	2.82	0.97–8.49	**0.001**	3.09	1.56–6.19
Proposed Ki67 grading	G1									
	G2a	0.646	0.77	0.25–2.30	**0.037**	6.01	1.30–44.15	0.482	1.34	0.59–3.11
	G2b	**0.048**	4.84	1.05–25.09	**0.004**	15.22	2.78–127.13	**0.002**	5.08	1.86–14.32
	G3	**0.017**	9.42	1.63–68.34	**0.001**	208.16	12.98–889.35	**< 0.001**	14.91	3.96–67.04


Bold values indicate statistical significance; *p* < 0.05

*PNI* Perineural invasion, *VI* Vascular invasion, *LNmet* Lymph Node metastasis, *Adverse outcome* liver/distant metastasis + disease-related death, *Tlow* T1 and T2, *Thigh* T3 and T4

## Discussion

This study, which is to our knowledge the first systematic pathologic analysis focusing on this issue, elucidates that, among resected PanNETs, by applying standardized counting methodology Ki67 index ≥ 10% identifies a significantly more aggressive and a metastasis-prone group and as such supports the evolving management protocols in the oncology literature that advocate managing this group differently.^3,7,9,24–35^ This category also stood the multivariate analysis along with lymph node metastasis and tumor size/stage.

It is important to point out that Ki67 index is a continuum; as such, it is plausible to pick essentially any cutoff and possibly find some prognostic value and associations. However, the current cutoff of 3–20%, which had been mostly extrapolated from G1 PanNETs proved to have virtually no discriminatory value, such that G1 and G2 PanNETs are currently mostly regarded together as one category (G1/2) for management purposes, leaving almost 95% of cases unstratified. In this study, 10% cutoff was selected and tested for two main reasons: (1) This cut off has recently begun to be employed in the oncology literature to select patients for therapy by somatostatin receptor analogues and others^3,7,9,24–35^; (2) The analysis of the primary cohort in our study in which Ki67 count was performed with special care revealed that with the “maximally selected rank statistics” method the proper cutoff was 12%. Considering this number is fairly close to the rule-of-thumb number of 10%, this figure was selected and indeed proved to have very striking discriminatory value.

Accordingly, evaluating G2 PanNETs that have a Ki67 index rate ≥ 10% separately as G2b (i.e., cases with Ki67 index of 10% to ≤ 20%), this group was found to exhibit significantly higher rates of metastasis than G2a group (i.e., Ki67 of 3% to < 10%). Other signs of aggressiveness also were significantly higher in the G2b group. Perhaps more importantly, these characteristics of G2b group was very similar to the G3 (>20%) group. Increasingly, the oncology literature encourages the consideration of therapy for any G3 PanNETs.^28,45^ Considering that the G2b cases were found to be very similar to G3 (both in the initial as well as the validation cohorts in this study), the approach applied to G3 cases may have to be considered for the G2b group, which needs to be further investigated by clinical trials. Clearly, if nothing else, this group is a candidate for closer follow-up. It should be reemphasized that his group is currently hidden in the wide G2 category and typically managed along with the G1 cases under the G1/2 umbrella; thus, their aggressiveness skips clinical attention.

The fact that G2b group is comparable to the G3 group suggests that the prognostic impact of Ki67 index peaks above 10% range but also starts to level off after a certain point probably as it approaches to the 20% range. In fact, in the primary cohort, the signs of aggressiveness were even higher in the G2b group than the G3 group. However, this may be related to the small number of cases in that initial cohort, because this difference mostly disappeared when combined with the validation cohort. Regardless, the findings are strongly indicative that G2b group defined is highly comparable to the G3 cases. It should be noted that, although well-differentiated PanNETs are viewed to be molecularly entirely distinct from PDNECs, recent studies are finding G3 cases to have some molecular overlaps with PDNECs^5,46^ with 35% of the cases showing p53 alteration. Considering G2b group shows aggressive behavior similar to G3, it will be important to analyze this group at the molecular level to see if it bears cases that show overlaps with (or ability to transition to) the PDNECs.^5^

It is important to reiterate that, in the current daily practice, the differences in aggressiveness between the G1 and the current broad G2 category (3–20%) is not viewed to be striking enough by the majority of the community to justify a differential treatment based on the grade alone.^7,25,47^ However, this study demonstrates that, when the characteristics of the G2b group is compared to G1, with the lymph node metastasis rate being several folds higher, and liver-distant metastasis rate of very compelling nine- to tenfold (58.5% vs. 6.1%), the significance of separating the G2b category and distinguishing it from the G1 becomes much more crucial.

These results also reemphasize some of the evolving practice recommendations for pathologists. First, the impression that “there is not much difference between a Ki67 of 4% and 19%” (all currently regarded G2), and “in fact, there is not much difference between G1 and G2 PanNETs” which in turn leads to the common application of eye-balling method in daily practice, is no longer valid. The striking differences in the behavior of G2a versus G2b group (which is even more dramatic when G2b is compared with G1) elucidated in this study necessitates more careful counting and numerical documentation of the Ki67 index. It should be reiterated that, after all, Ki67 index is a continuous variable, and that 9% (regarded G2a in this study) is probably not really that different than 11% (regarded G2b). As in any grading or staging system, there are imperfections in the cutoff regions. For this reason, in our opinion, documentation of the specific count (not only the grade) in the surgical pathology report is warranted. This would allow the management team to assess other findings, for example, the presence of other risk factors such as the highly infiltrative pattern^16^ or oncocytic phenotype,^12^ and incorporate age, patient expectation/choices and comorbidities to determine the course of action putting all these factors together. For this reason, we believe it is crucial to provide a specific number for Ki67 index, and we also recommend reporting of infiltration pattern and phenotypic classification, all of which altogether could aid in the final clinical decision making, especially for the cases that stand on the fence. Of note, despite the acknowledged shortcomings in applying cutoffs to a continuous process, at the same time, as in any grading and staging system, a cutoff to stratify the patients also is needed for Ki67. In this study (in which the hard stop cutoff was found to be 12% by the “maximally selected rank statistics”) when combined with the evolving impression in oncology literature regarding 10%, points to this rule of thumb number of 10% as the most practical and valid cutoff.^24–27^ As such, all of this warrants the creation of a G2b category.

The findings in this study will have significant impact on the treatment of PanNET patients. For management of tumors that have already been resected, it has not been clear which radiologic test to employ, how soon, and with which frequency. The rates of metastasis elucidated in this study for G2b cases (with three quarters of this group showing lymph node metastasis, and almost two thirds showing liver/distant metastasis, the latter 8 times more frequent than in the G1 cases), clearly indicates that this group warrants close follow up at minimum. In fact, depending on the age of the patient and other factors, the eligibility of G2b cases to a management that is similar to G3 (for which somatostatin analogues and other therapies are increasingly being employed as adjuvant treatment) may have to be considered, although, of course, this requires further studies.^24–28^ However, with a rate > 60% showing liver/distant metastasis, for this group, it is clear that surveillance protocols need to be modified; at minimum frequent liver radiograms are warranted for the G2b group, probably starting with 3-month intervals and extending them as time goes by, which is what we currently recommend for our patients. In contrast, for a G1 PanNET, which overall has less than 10% liver/distant metastasis rate, the surveillance may not have to be that intense, and an adjuvant therapy would obviously not be justifiable.

The results of this study also has significant implications on the management of PanNETs that are smaller than 2 cm. Currently, watchful waiting is considered as an option of these cases, regardless of the grade.^10,11^ Indeed, in this study, for < 2 cm tumors that are G1, the liver/distant metastatic rate at the follow up period was < 2%, and for even G2a, it was < 10%. However, when it came to the proposed G2b group, the rate of metastasis was 50% even in these small tumors. Granted this constitutes a relatively small percentage of the cases (7% of < 2 cm PanNETs are G2b and 3% G3), but nevertheless it is not a trivial number. These data indicate that if such a case is encountered, resection should be considered strongly.

Naturally, these approaches will have to be modified as more data comes out for the different subgrades, but in the absence of any specific data at this time, the approaches described above are justifiable based on this data and current literature. Clinical trials are warranted to consolidate new guidelines for these groups.

## Data Availability

The datasets used and/or analyzed during the current study are available from the corresponding author on reasonable request.
